# Clinical characterization of immunocompetent patients with *Scedosporium* detected in respiratory samples: A case series

**DOI:** 10.1016/j.rmcr.2025.102256

**Published:** 2025-07-07

**Authors:** Yasuhiro Ito, Seiichi Miwa, Akira Watanabe, Masahiro Shirai

**Affiliations:** a Department of Respiratory Medicine, NHO Tenryu Hospital, Hamamatsu, Japan; b Division of Clinical Research, Medical Mycology Research Center, Chiba University, Chiba, Japan

**Keywords:** *Scedosporium*, Immunocompetent, Infection, Colonization, Bronchiectasis, MAC pulmonary disease

## Abstract

Although *Scedosporium* is increasingly recognized in pulmonary infections, the optimal management of immunocompetent patients with *Scedosporium* detected in respiratory samples is poorly understood. This study aimed to characterize the clinical features of immunocompetent patients with *Scedosporium* detected in respiratory specimens. We retrospectively reviewed cases from 2014 to 2022 at our hospital. Eight elderly, immunocompetent female patients were identified, all presenting with bronchiectasis on chest imaging. Notably, seven (87.5 %) had a history of pulmonary *Mycobacterium avium* complex (MAC) disease. Based on their clinical courses, seven patients were classified as having colonization. One patient, diagnosed with infection, was treated with voriconazole, and her clinical symptoms stabilized without negative conversion of sputum cultures. Our findings suggest that bronchiectasis, particularly when linked with pulmonary MAC disease, may serve as an important underlying condition for *Scedosporium* colonization or infection in immunocompetent individuals. In the majority of cases, *Scedosporium* is indicative of colonization. However, distinguishing between colonization and infection remains a critical challenge for respiratory physicians.

## Highlights

• All immunocompetent patients had bronchiectasis on chest CT.
• Bronchiectatic MAC-PD is key to *Scedosporium* isolation in immunocompetent patients.
• *Scedosporium* in respiratory samples of immunocompetent people suggests colonization.
• Distinguishing *Scedosporium* colonization from infection remains a crucial challenge.

## 1 Introduction

*Scedosporium* is a filamentous fungus found worldwide that can thrive in various environmental settings, including soil, water, and decaying vegetation [1,2]. It primarily infects immunocompromised individuals, sometimes leading to severe conditions like pneumonia and disseminated infections affecting multiple organs [3,4].

Immunocompetent individuals can occasionally be infected by *Scedosporium* through inhalation of spores from the environment, particularly following near-drowning incidents or in the presence of underlying lung conditions [[5], [6], [7], [8]]. A well-known instance of the former is the "Tsunami lung" cases following the Great East Japan Earthquake on March 11, 2011 [9]. Regarding the latter, *Scedosporium* can cause lung infections in patients with pulmonary tuberculosis, bronchiectasis, or chronic obstructive pulmonary disease (COPD) [[5], [6], [7], [8]].

Pulmonary *Scedosporium* infections are generally rare in immunocompetent individuals [[5], [6], [7], [8]]. However, *Scedosporium*'s notable resistance to many conventional antifungal treatments poses significant challenges for respiratory physicians [10,11]. These challenges include determining whether the detected *Scedosporium* are causing an infection or merely colonizing the respiratory tract.

In this study, we aim to clarify the clinical characteristics of immunocompetent patients in whose respiratory samples *Scedosporium* were detected.

## 2 Materials and methods

From January 2014 to December 2022, we retrospectively examined consecutive patients who attended our institution with detection of *Scedosporium* species in their respiratory specimens obtained by sputum or bronchoscopy.

The fungal examination method involved 10μg of the specimen onto potato dextrose agar supplemented with chloramphenicol (EIKEN CHEMICAL CO., LTD) and incubating at 35 °C for up to 10 days. Upon the growth of filamentous fungi, the hyphae were observed under a microscope to identify the fungal species.

We collected patient data from medical records, including symptoms, laboratory findings, radiographic findings, and clinical courses. Cases were classified by multiple pulmonologists as either "colonization" when *Scedosporium* was present without associated symptoms, or "infection" when *Scedosporium* was detected more than twice with accompanying clinical symptoms. The study protocol was approved by the Ethical Committee of NHO Tenryu Hospital. The need for patient approval and/or informed consent was waived due to the retrospective nature of the study.

## 3 Results

Eight patients had *Scedosporium* detected in their respiratory samples. Identification method used was sputum cultures in seven patients and bronchial lavage cultures in two patients. Clinical characteristics at first isolation are shown in Table 1. All patients were elderly immunocompetent female (range 71–90 years old). One patient (case 3) had a history of hepatocellur carcinoma in complete remission of following surgery, as well as RNA-negative chronic hepatitis C without cirrhosis. None of the patients were smokers or used oral or inhaled steroids. As for underlying pulmonary disease, seven patients had pulmonary *Mycobacterium avium* complex (MAC) disease, and one had bronchiectasis. Regarding chest imaging, bronchiectasis was observed in all, cavities in three, and a fungal ball in one. All patients exhibited symptoms such as cough and sputum production. Only one patient (case 3) had elevated white blood cell counts, which were attributed to empyema caused by MAC. Two patients had elevated serum β-D-glucan levels. Five patients had a history of *Aspergillus* isolation in their respiratory samples, while two had *Pseudomonas aeruginosa* identified in theirs. Concerning the state of pulmonary MAC disease, two patients were remission, three had relapsed, two had refractory diseases. At that time, four patients received low-dose erythromycin for its anti-inflammatory effects, and two patients received itraconazole (ITCZ) for *Aspergillus* isolation.

**Table 1:** Clinical characteristics at first isolation of *Scedosporium*.

No	Age(years) /Gender	Underlying disease	Underlying Respiratory disease	Chest imaging			Respiratory samples	WBC (/μL)	β-DG (pg/mL)^a^	History of isolation		State of MAC disease	Treatment
										Aspergillus	Psedomonas aeruginosa		
				BE	Cavity	Fungus ball							
1	87/F	HT, Paf	MAC	+	–	–	sputum	6130	9.9	–	–	Relapse	–
2	78/F	HT, HL	BE	+	–	–	sputum	4430	46.3	–	+	–	–
3	75/F	CH(C), HCC surgery	MAC	+	+	+	sputum	11170	11.1	–	–	Refractory	EM
4	90/F	DM	MAC	+	+	–	sputum	5500	<5.0	+	–	Relapse	–
5	79/F	HT	MAC	+	–	–	sputum	7330	22.2	+	–	Refractory	EM, ITCZ
6	77/F	HT	MAC	+	–	–	sputum	5040	<5.0	+	–	Relapse	–
7	74/F	DM, HT	MAC	+	–	–	sputum	3540	18.7	+	+	Remission	EM, ITCZ
8	71/F	Hypothyroidism, Dyspepsia	MAC, Nocardiosis	+	+	–	BAL, sputum	4610	<5.0	+	–	Remission	EM, ITCZ

a Reference values < 20, Abbreviations: BE, bronchiectasis; WBC, white blood cell; β-DG, β-D-glucan; MAC, *Mycobacterium avium* complex; HT, hypertension; Paf, paroxysmal atrial fibrillation; CH(C), chronic hepatitis C; HCC, hepatocellular carcinoma; DM, diabetes mellitus; BAL, Bronchoalveolar Lavage; EM, erythromycin; ITCZ, itraconazole.

Table 2 shows the clinical courses after first isolation of *Scedosporium*. *Scedosporium* were detected multiple times in four patients (case 1, 4, 5, 8). Based on their clinical courses, we classified seven patients as having colonization and one patient as having an infection. In cases 1 and 4, since *Scedosporium* was not spontaneously detected without antifungal treatment, these cases were regarded as colonization. In case 5, symptoms were initially attributed to *Mycobacterium abscessus*, but the attending physician could not rule out the possibility of *Scedosporium* infection and administrated voriconazole (VRCZ). However, despite the clearance of *Scedosporium*, the patient's condition continued to deteriorate, leading us to diagnose the patient with colonization.

**Table 2:** Clinical course after first isolation of *Scedosporium*.

No	Number of detection	Duration of detection (months)	Clinical course	*Scedosporium* Status		Prognosis
				Culture	Colonization vs. Infection	
1	10	48	Treatment for relapsed MAC	Spontaneous negative conversion	Colonization	Alive
2	1		Treatment for occurrence of TB and Aspergillosis		Colonization	Alive
3	1		Treatment for complicated MAC empyema		Colonization	Dead (due to MAC empyema)
4	3	17	Observation	Spontaneous negative conversion	Colonization	Alive
5	4	23	Treatment for MAC, MAB, and *Scedosporium*	Negative conversion	Colonization	Dead (due to MAB)
6	1		Treatment for relapsed MAC		Colonization	Alive
7	1		Treatment for Aspergillosis and relapsed MAC		Colonization	Alive
8	10	>12	Completion of Nocardiosis treatment followed by treatment for *Scedosporium*	Persistent positivity	Infection	Alive

Abbreviations: MAC, *Mycobacterium avium* complex; TB, tuberculosis; MAB, *Mycobacterium abscessus* complex.

Representative cases are detailed below.

Case 1 Colonization

**Fig. 1:**
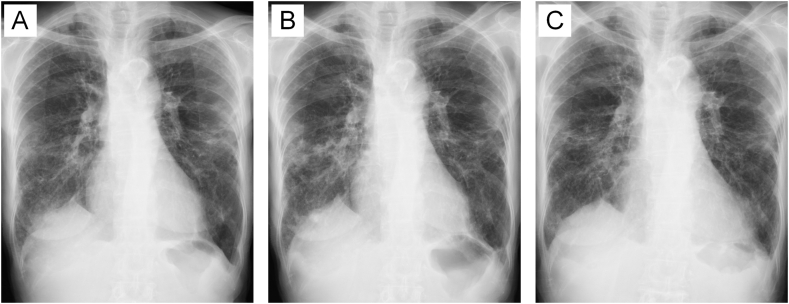
Clinical course of Case 1. Compared to six months prior [A], chest X-ray at the initial detection of *Scedosporium* showed increased infiltration in the right lung [B]. Two years and eight months later following re-treatment for MAC-PD, the chest X-ray demonstrated improvement despite persistent positive cultures for *Scedosporium* [C].

A female patient who had completed two full courses of treatment for MAC pulmonary disease was under observation (Fig. 1A). However, chest X-rays showed worsening (Fig. 1B) compared to six months prior. At that time, both *Scedosporium* and MAC were detected in her sputum, prompting a re-treatment for MAC, which led to improvement on the X-rays (Fig. 1C) though symptoms remained unchanged. *Scedosporium* was detected ten times over four years but eventually became undetectable spontaneously.

Case 8 Infection

**Fig. 2:**
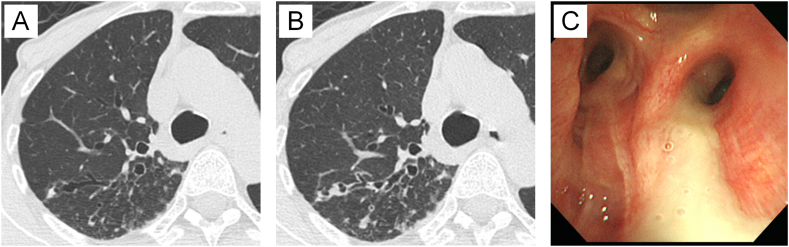
Clinical course of Case 8. Compared to nine months prior [A], chest computed tomography (CT) at the first detection of *Scedosporium apiospermum* showed worsened infiltration and thickening of the bronchial walls [B]. Bronchoscopy revealed a large amount of yellow sputum from right B2 bronchus [C], which was identified as *Scedosporium apiospermum*.

A female patient developed pulmonary nocardiosis following treatment for MAC pulmonary disease. Despite receiving treatment for pulmonary nocardiosis, her cough and sputum production worsened, and she began experiencing hemoptysis. Compared to nine months earlier (Fig. 2A), chest computed tomography (CT) revealed bronchial wall thickening and worsening of infiltration (Fig. 2B). Bronchoscopy revealed the presence of *Scedosporium* (Fig. 2C), identified as *Scedosporium apiospermum* (*S. apiospermum*) through internal transcribed spacer sequence (ITS) analysis. Her symptoms persisted, and *Scedosporium* was detected not only through bronchoscopy but also in sputum, while MAC and *Nocardia* were not, leading to a diagnosis of *Scedosporium* infection. The patient was treated with VRCZ for the infection, but initially, her symptoms and inflammatory markers worsened. After increasing the VRCZ dose, her symptoms and inflammatory markers improved, though *S. apiospermum* continued to be persistently detected in her sputum. The drug susceptibility profile of *S. apiospermum* isolated from the patient is shown in Table 3.

**Table 3:** Result of antifungal susceptibility test.

Antifungal agent	MIC (μg/ml)
Micafungin (MCFG)	>16
Caspofungin (CPFG)	8
Amphotericin B (AMPH-B)	2
Itraconazole (ITCZ)	>8
Voriconazole (VRCZ)	2

Abbreviation: MIC, minimum inhibitory concentration.

## 4 Discussion

*Scedosporium* is an increasingly recognized fungus due to its involvement in difficult-to-treat infections, affecting not only immunocompromised but also, less commonly, immunocompetent individuals. It is critical for respiratory physicians to distinguish between colonization and true infection by *Scedosporium*, especially in immunocompetent patients. Although several case reports have described pulmonary *Scedosporium* infections in immunocompetent individuals [[5], [6], [7], [8]], there are no comprehensive studies on the prevalence of colonization in this population. In our study, we identified *Scedosporium* in their respiratory samples of eight patients, diagnosing seven (87.5 %) with colonization. All of these patients had bronchiectasis as an underlying lung condition, and seven had a history of pulmonary MAC disease. Notably, among 350 patients with a history of pulmonary MAC disease who attended our hospital during the study period, *Scedosporium* was detected in 2 % (7/350). All patients presented with respiratory symptoms attributed to their underlying pulmonary MAC disease and bronchiectasis, which complicated the differentiation between colonization and infection at the time of initial detection of *Scedosporium*. Careful follow-up is essential to definitively differentiate between these two clinical entities.

While *Scedosporium* infection in immunocompromised patients is associated with high mortality [4,12], data on the prognosis in immunocompetent individuals are limited. A systematic review of pulmonary *Scedosporium* infections in immunocompetent patients reported a mortality rate of 12.5 % [8]. In our study, one patient diagnosed with infection showed clinical improvement with VRCZ treatment, despite not achieving negative culture conversion. Although the reported mortality rate is not negligible, based on our experience, we believe that when *Scedosporium* is detected in respiratory specimens of immunocompetent individuals, it is crucial to accurately determine whether it represents an infection or colonization, rather than immediately initiating antifungal treatment, unless the patient's general or respiratory condition is critical. Distinguishing between infection and colonization is often challenging, as there is no universally accepted definition to differentiate the two in clinical settings. In our study, the classification was based on the presence of consistent clinical symptoms and repeated isolation of *Scedosporium*. Although inflammatory markers such as white cell count and C-reactive protein, as well as fungal biomarkers like β-D-glucan were reviewed in all patients, we did not consider them central to the diagnostic classification, as coexisting pulmonary MAC disease, *Aspergillus*, or *Pseudomonas aeruginosa* infection could influence these markers. Therefore, clinical judgment must be made holistically, taking into account the overall disease course, including clinical presentation, radiographic findings, and microbiological data. We strongly agree that careful longitudinal follow-up is essential, especially in borderline cases, to detect clinical changes over time and to guide antifungal treatment appropriately.

In cystic fibrosis (CF) patients, *Scedosporium* is the second most commonly detected fungus after *Aspergillus* [13,14]. Possible risk factors for *Scedosporium* isolation in these patients include prior isolation of *Pseudomonas*, the use of parenteral antibiotics, a history of allergic bronchopulmonary aspergillosis, and previous administration of ITCZ [15,16]. Among our eight patients, two had *Pseudomonas*, five had *Aspergillus*, and two had received ITCZ for the treatment for aspergillosis. *Scedosporium* is known to have poor susceptibility to ITCZ and amphotericin B [10]. Therefore, when treating aspergillosis in patients with bronchiectasis or pulmonary MAC disease, it is important to recognize that ITCZ is ineffective against *Scedosporium* and may contribute to its isolation.

The limitations of this study include its retrospective nature and the small sample size from a single institution. Additionally, the frequency of sputum collection and radiographic imaging was not standardized.

## 5 Conclusion

Bronchiectasis, particularly in the context of pulmonary MAC disease, may be a significant underlying condition for *Scedosporium* colonization or infection in immunocompetent individuals. In most cases where *Scedosporium* is isolated in respiratory specimens from immunocompetent patients, it is likely to represent colonization. However, distinguishing colonization from infection remains a critical task for respiratory physicians.

## CRediT authorship contribution statement

**Yasuhiro Ito:** Writing – original draft, Investigation, Data curation, Conceptualization. **Seiichi Miwa:** Writing – review & editing, Writing – original draft. **Akira Watanabe:** Data curation. **Masahiro Shirai:** Supervision.

## Declaration of generative AI and AI-assisted technologies in the writing process

During the preparation of this work the authors, who are non-native English speakers, used ChatGPT-4o in order to revise sentences and enhance readability. After using this tool, the authors reviewed and edited the content as needed and take full responsibility for the content of the publication.

## Funding sources

None.

## Declaration of competing interest

None.
